# Vascular segmental exclusion for recurrent vulvar squamous cell carcinoma invading the femoral vessels

**DOI:** 10.1016/j.jvscit.2025.101753

**Published:** 2025-02-19

**Authors:** Andrew W. Schwartz, Masoud Azodi, Jonathan Cardella, Cassius Iyad Ochoa Chaar

**Affiliations:** a Yale School of Medicine, New Haven, CT; b Department of Obstetrics and Reproductive Sciences, Gynecologic Oncology Section, Yale School of Medicine, New Haven, CT; c Division of Vascular Surgery and Endovascular Therapy, Department of Surgery, Yale School of Medicine, New Haven, CT

**Keywords:** Axillopopliteal bypass, Gynecologic malignancy, Oncovascular surgery

## Abstract

A 55-year-old woman presented with a recurrent left inguinal necrotic mass caused by locally invasive vulvar carcinoma. Computed tomography of the abdomen pelvis showed compression of the femoral vein and superficial femoral artery. The patient underwent a left axillary to popliteal artery bypass with left superficial femoral artery embolization in preparation for possible wide resection. Subsequent resection was aborted because of extensive local invasion and rapid spread. Three-month follow-up revealed a patent graft. The patient died after 4 months from progression of oncologic disease and an unresectable tumor in the groin that eroded through the blood vessels without bleeding or lower limb ischemia.

Vascular reconstruction in aggressive gynecologic malignancy increases the likelihood of achieving complete resection and preserving flow to critical organs, thereby improving life expectancy and maintaining quality of life.^1^^,^^2^ Vulvar squamous cell carcinoma (VSCC) represents 3% to 5% of all gynecologic malignancies with 25% to 30% of patients presenting with lymph node involvement.^3^ Local recurrence occurs almost one-third of the time after resection and radiation.^4^ Recurrence may cause infection and vascular invasion, increasing risk of limb loss and catastrophic hemorrhage.^5^ The axillary to popliteal artery bypass may be a useful revascularization strategy in patients with infected, irradiated groins by enabling limb-salvage and radical resection.^6^, ^7^, ^8^ This is a case of a patient with a recurrent, chronically infected inguinal necrotic mass and femoral vessel invasion due to VSCC who underwent an axillary to popliteal artery bypass with embolization of the femoral artery. The patient’s next of kin consented for publication, given the patient is deceased.

## Case report

**Fig 1:**
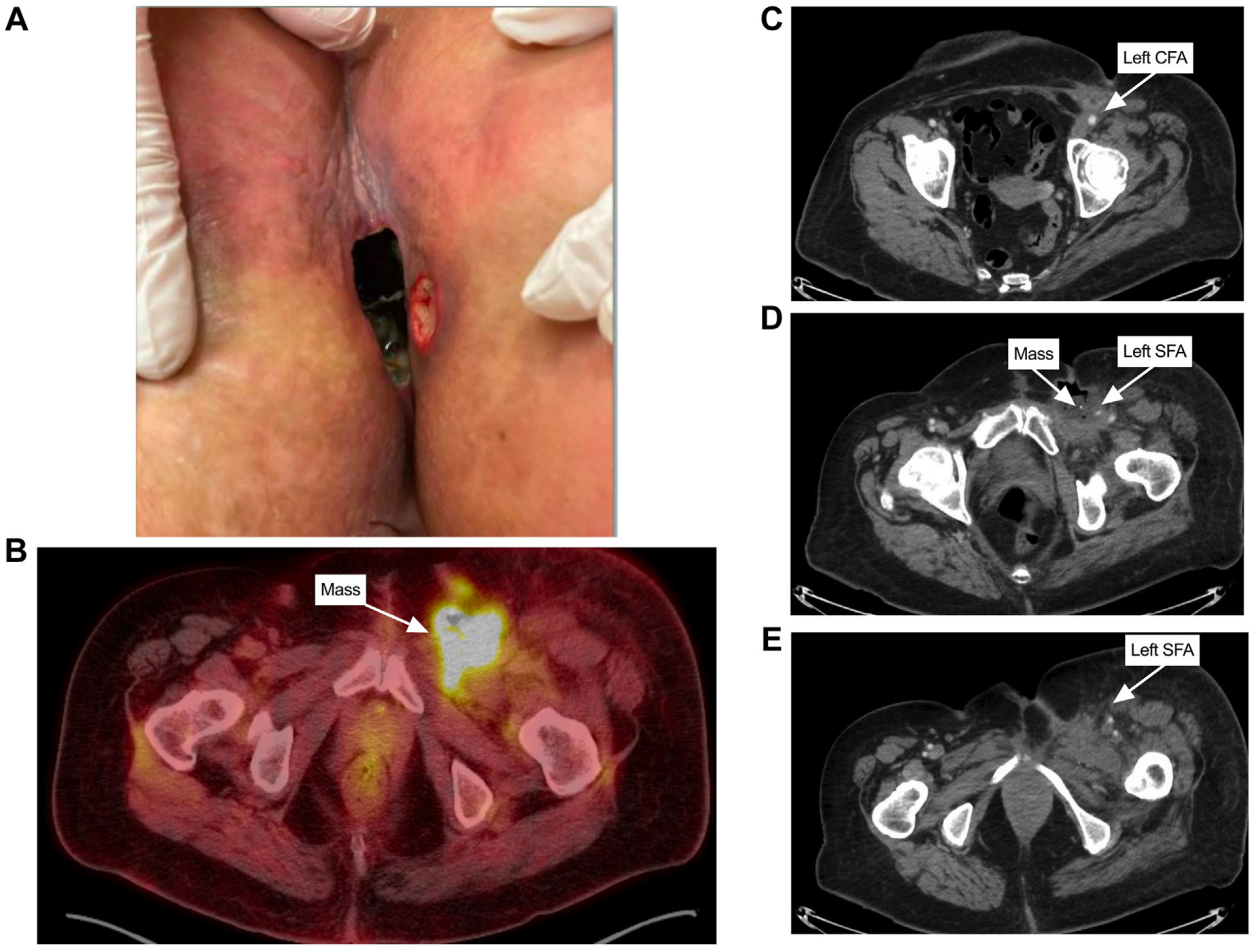
Preoperative wound and imaging. **(A)** Left groin infected and necrotic wound at presentation. **(B)** Preoperative positron emission tomography scan at the level of the left groin mass. **(C)** Axial cross-sections from the preoperative computed tomography (CT) angiography at the level of the **(C)** common femoral artery (*CFA*), **(D)** left groin necrotic mass, **(E)** superficial femoral artery (*SFA*) below the level of the mass showing compression of the left SFA.

A 55-year-old woman with obesity (body mass index, 31.9 kg/m^2^) and recurrent VSCC presented with an open, expanding, chronically infected, necrotic lymph node recurrence that progressed after left groin debulking 4 months prior despite broad microbial coverage with trimethoprim/sulfamethoxazole (Fig 1, *A*). She underwent a radical partial vulvectomy with left inguinal lymph node excision 13 months prior and subsequent 5 weeks of chemo-radiation therapy with cisplatin. She progressed despite multiple cycles of cytotoxic and biological chemotherapy.

She was ambulatory and functionally independent, had palpable left dorsalis pedis/posterior tibialis (DP/PT) pulses, and stable left lower extremity edema from mass effect on the left common femoral vein (FV) assessed 2 months prior. She had started prophylactic apixaban 2.5 mg twice daily initiated by the hematology service. FV stenting was avoided, given the risk of stent thrombosis from inguinal compressive forces. She was on aspirin 81 mg for coronary stents. Positron emission tomography scan revealed a left inguinal hypermetabolic region (Fig 1, *B*). Computed tomography (CT) revealed a centrally necrotic mass impeding on the left FV and superficial femoral artery (SFA) (Fig 1, *C-E*). Tissue cultures were positive for mixed flora, and she was admitted on intravenous vancomycin and piperacillin/tazobactam.

Invasion of the femoral vessels raised concern for arterial blowout and acute arterial occlusion from mass effect on the SFA. After a multi-disciplinary discussion between vascular surgery, medical oncology, and gynecologic oncology, the decision was to perform vascular segmental exclusion (VSE) of the femoral artery with an axillary to popliteal artery bypass to maintain limb perfusion and embolization of the femoral artery to prevent hemorrhage in preparation for possible wide resection and debridement. Alternative surgical approach with in situ reconstruction and complex plastic surgery coverage or obturator bypass was entertained but thought to be less favorable. With the initial plan to do en bloc resection, any vascular reconstruction would be at risk, as the soft tissue coverage would be suboptimal.

**Fig 2:**
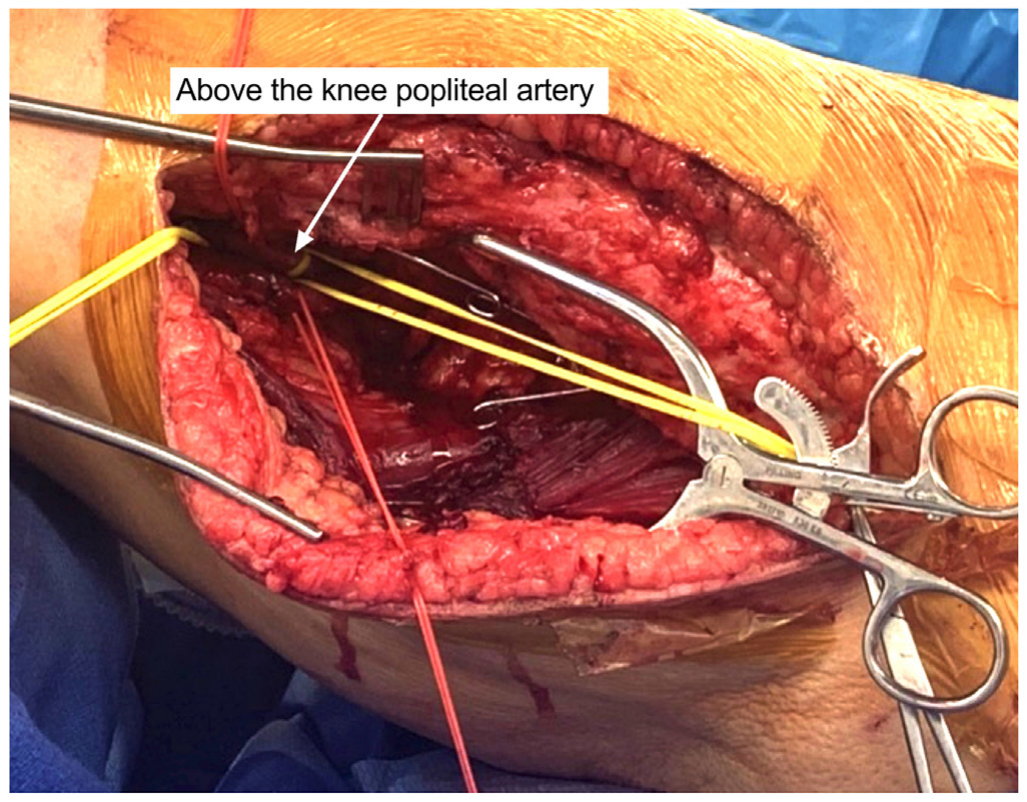
Lateral exposure of the left popliteal artery.

**Fig 3:**
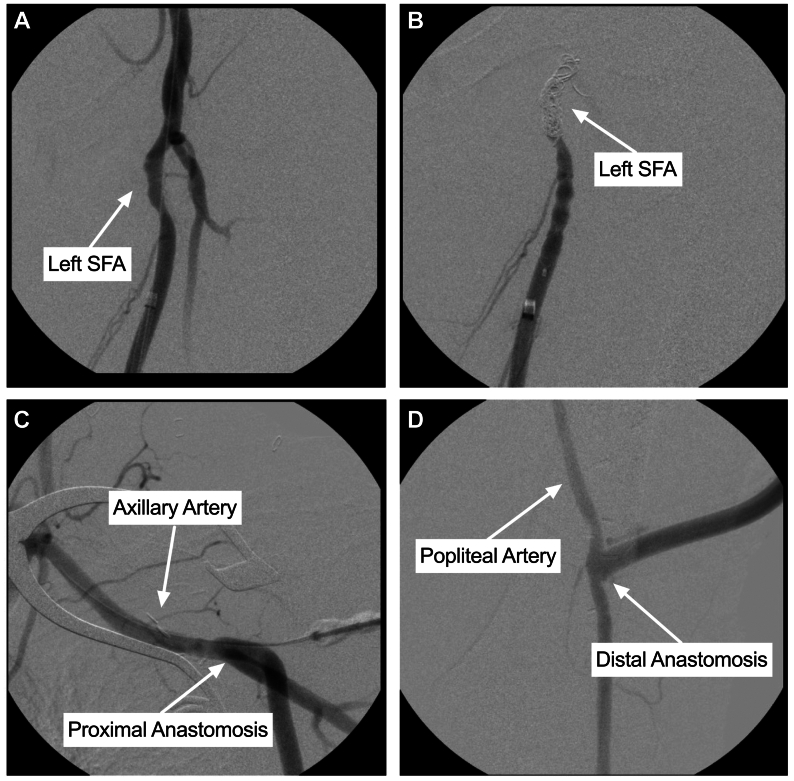
Intraoperative angiogram showing compression of the left superficial femoral artery (*SFA*) by the mass **(A)** left common femoral artery (CFA) and SFA post-coil embolization and plug **(B)**, proximal anastomosis at the axillary artery **(C)**, and distal anastomosis at the popliteal artery **(D)**.

Under general anesthesia, the left axillary artery was exposed at the infraclavicular region, and the left popliteal artery was exposed at the lateral thigh (Fig 2). Both arteries were controlled using vessel loops. Endovascular access was obtained through direct left popliteal artery puncture, and an angiogram showed compression of the proximal SFA (Fig 3, *A*) with flow to the distal SFA with three-vessel runoff. A 14-mm Amplatzer plug (Abbot) was placed in the common femoral artery, a 12 mm × 60 cm coil (Penumbra Inc) was placed from the common femoral artery to the proximal SFA, and a 10-mm Amplatzer plug was placed in the proximal SFA. Embolization was completed, and angiogram showed negligible flow around the coils (Fig 3, *B*). Two 8-mm ringed PTFE grafts (Gore Medical) were needed to reach the length from the axillary to the popliteal artery. The grafts were tunneled subcutaneously and sutured at a lateral flank incision. The tunnel crossed lateral to the anterior superior iliac spine. Anastomoses were completed at the axillary and popliteal artery (Fig 3, *C* and *D*). After wound closure, gynecologic oncology found the resection was limited by dense pelvic ramus adherence, resulting in local debridement and washout.

**Fig 4:**
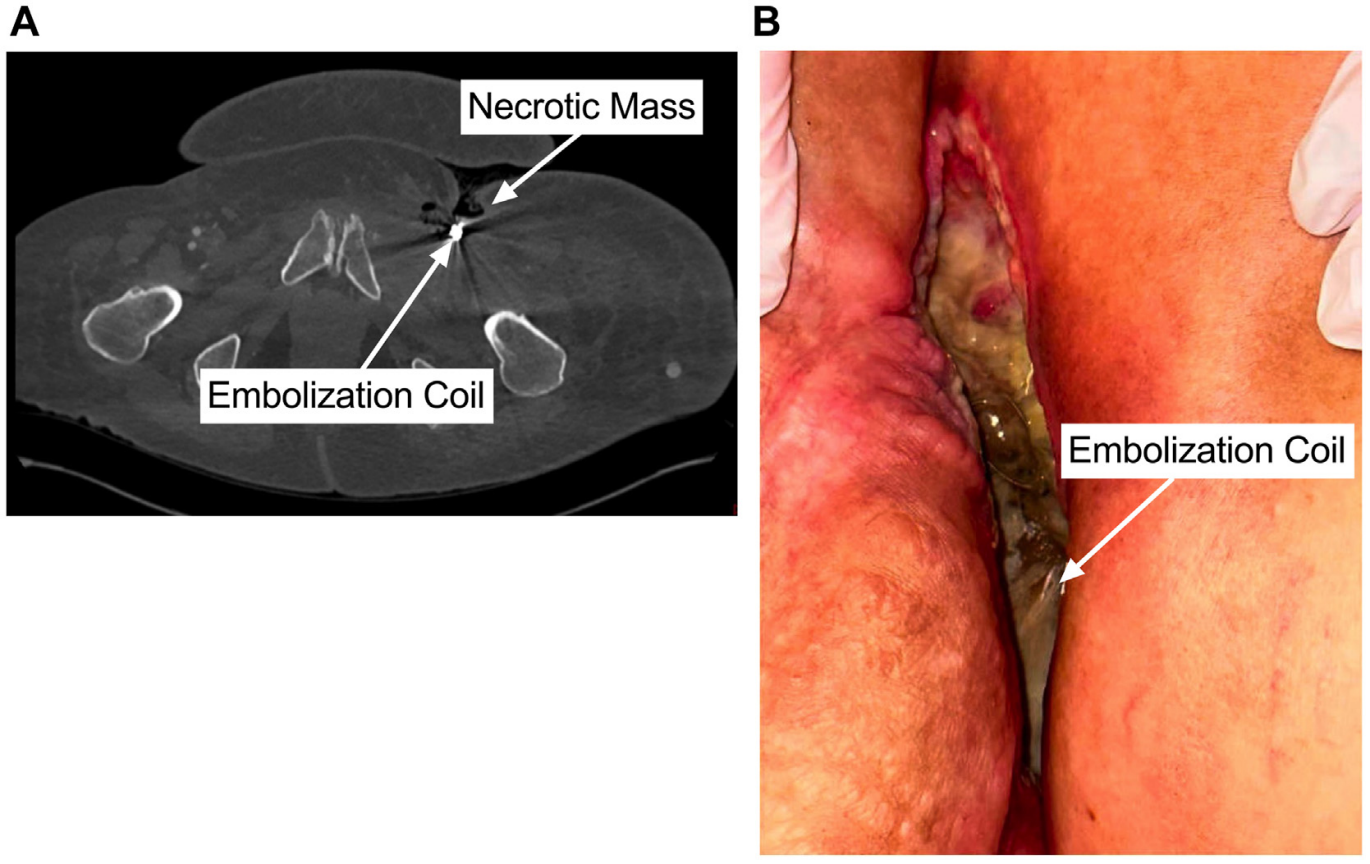
Postoperative left groin embolization coil erosion. **(A)** Three-month postoperative computed tomography (CT) angiography showing erosion of the embolization coil at the level of necrotic mass. **(B)** Three-month postoperative left groin with embolization coils eroded through the femoral artery without hemorrhage.

Postoperatively, the patient had palpable DP/PT pulses. She was on a heparin drip and transitioned to apixaban 5 mg for a left common FV deep venous thrombosis. She restarted aspirin 81 mg. She was placed on daptomycin and piperacillin/tazobactam for operative wound cultures positive for vancomycin-resistant *Enterococcus faecium*. She was discharged home on postoperative day 10 with home nursing. She maintained ambulation with a walker. After three rounds of chemotherapy, she was admitted 3 months postoperatively with intractable left groin pain, worsening left lower extremity edema, and palpable DP/PT pulses. Her CT of the abdomen/pelvis showed increased mass size and gas near the pubic ramus with cortical erosion consistent with oncologic disease progression (Fig 4, *A*). On CT, the bypass remained patent, but coils were visible at the left groin (Fig 4, *B*). Following a joint goals of care discussion, she was placed on hospice then passed away 4 months postoperatively without bleeding or ischemia to the left limb.

## Discussion

This case highlights a rare, locally invasive, infected, and necrotic VSCC invading the femoral vessels. VSE of the femoral artery prevented hemorrhage, and an axillo-popliteal extra-anatomic bypass prevented limb ischemia. The surgery was performed as a bridge to radical resection to prolong life and maintain function. Although the patient succumbed to cancer, she was provided a chance at resection that would improve survival and possibly provide remission. This strategy was effective in preventing hemorrhage and limb ischemia in this context and may be used in similar oncologic settings with major blood vessel invasion as a bridge to resection and debulking. If the mass was deemed unresectable, the more prudent approach would be to address complications as they arise rather than performing prophylactic surgery, as life expectancy would be limited.

The axillary to popliteal artery bypass may be favored for those with prior irradiation and extensive groin infection.^8^^,^^9^ Primary patency of the axillary to popliteal artery bypass in a series of studies ranged from 64% to 86% at 1 year.^10^ A case report of VSCC groin recurrence described the feasibility of the axillo-popliteal artery bypass in a similar context, although it differs as the femoral artery was resected rather than embolized.^8^ An endovascular approach to VSE was favored in this case to minimize dissection in a chronically infected and irradiated groin. Embolization was also favored to decrease risk of hemorrhage and ease resection. The axillo-popliteal bypass was the preferred option in this case as other revascularization options such as in-line bypass or obturator bypass could have subjected the patient to vascular reconstruction in an infected, irradiated field. Soft tissue coverage of vascular reconstruction in the medial inguinal area or obturator area would have been challenging. VSE with distal revascularization would be the preferred strategy if the mass was resectable, as it provides palliation for a young patient expected to regain independent function. This patient had threatened femoral artery blow-out given the encasement of the femoral vessels, necessitating urgent intervention.^11^^,^^12^ For the above-the-knee popliteal artery, a lateral approach avoids graft kinking in a patient with elevated body mass index.^13^

## Conclusions

Overall, locally invasive VSCC with involvement of the femoral artery treated with extra-anatomic vascular reconstruction and coil embolization reduces the risk of catastrophic hemorrhage, provides limb salvage, and gives patients an opportunity for definitive resection and possible remission of their malignancy while providing palliation. This approach should be considered for future patients.

## Funding

None.

## Disclosures

None.
